# An Exceptional Mobility Group Among Older Adults: Protocol for the Super Movers Study

**DOI:** 10.2196/104520

**Published:** 2026-10-30

**Authors:** Chathathayil Mohammedali Shafeeque, Kelly Cotton, Sanish Sathyan, Emmeline I Ayers, Valerie Nunez, Uros Marusic, Erica F Weiss, Daliah Ross, Jie Yang, Helena M Blumen, Jeannette R Mahoney, Joe Verghese

**Affiliations:** 1 Department of Neurology, Renaissance School of Medicine, Stony Brook Medicine, 101 Nicolls Rd, Stony Brook, NY, 11794, United States, 1 631-216-9370; 2 Department of Molecular Biology, Kannur University, Kannur, Keralam, India; 3 Institute for Kinesiology Research, Science and Research Centre Koper, Koper, Slovenia; 4 Department of Health Sciences, Alma Mater Europaea University, Maribor, Slovenia; 5 Department of Neurology, Albert Einstein College of Medicine, Bronx, NY, United States; 6 Department of Psychiatry and Human Behavior, Warren Alpert Medical School of Brown University, Providence, RI, United States; 7 Department of Family, Population & Preventive Medicine, Renaissance School of Medicine, Stony Brook University, Stony Brook, NY, United States

**Keywords:** gait resilience, super movers, neuroimaging, mobile EEG, multisensory integration, blood-based biomarkers, healthy aging, mobile electroencephalogram

## Abstract

**Background:**

Gait dysfunction is a preclinical marker of Alzheimer disease (AD) and related dementias (ADRD), often emerging years before clinical diagnosis. While much of the existing literature has focused on the pathological mechanisms driving motor and cognitive decline, the neural and biological substrates that confer resilience against such decline remain poorly understood. Individuals who maintain exceptional gait performance into advanced age, termed Super Movers, represent a unique phenotype of resilient aging. Understanding the biological profile of these individuals offers a unique and largely untapped window into the mechanisms underlying healthy aging and resilience to dementia.

**Objective:**

This study aims to identify the structural, functional, neurophysiological, and molecular brain characteristics that distinguish older adults with exceptional mobility from their usual and slow-moving peers, and to determine whether these characteristics predict cognitive and physical decline over time. We hypothesize that the interplay between protective and pathological mechanisms in aging gives rise to structural, functional, neurophysiological, and molecular brain changes that manifest along a continuum from resilient gait to gait dysfunction.

**Methods:**

We aim to enroll 350 adults aged 65 years and older from community and clinic settings. Participants will be classified by gait speed into fast, usual, and slow movers using age- and sex-specific thresholds; fast movers aged 80 years and older will be designated as Super Movers. All participants will undergo comprehensive assessments at baseline, including brain imaging, mobile electroencephalogram (EEG) recorded during walking, multisensory integration (MSI), comprehensive blood profiling, blood-based markers of neurodegeneration, epigenetic and proteomic aging clocks, and detailed cognitive testing. Follow-up assessments will be conducted annually in person and every 6 months by telephone.

**Results:**

This study was funded by the National Institute on Aging (R01AG089096; PI: Joe Verghese) and has received Institutional Review Board (IRB) approval from Stony Brook University. Enrollment is currently underway.

**Conclusions:**

Fast and Super Movers are expected to demonstrate distinct neural substrates, EEG activation signatures, and favorable biomarker profiles relative to usual and slow movers. By shifting the scientific focus from the biology of gait decline toward the biology of gait resilience, this protocol is uniquely positioned to advance our understanding of healthy aging and inform novel strategies for dementia prevention.

## Introduction

### Background

Physical mobility is important for healthy aging. The ability to remain mobile in aging supports better health, longevity, and greater independence. Walking is a fundamental expression of mobility, and gait performance offers meaningful insight into current functional capacity and future mobility trajectories. A stable and efficient gait reflects preserved balance, coordination, and physiological reserve-body’s capacity to maintain function and respond to physiological stressors. Slow gait, by contrast, may signal underlying health problems and has been associated with falls, disability, hospitalization, and cognitive decline in older adults [1-6]. As one of the most informative indicators of overall health status in later life, gait assessment is a vital component of strategies to promote healthy aging [7-9]. Early subtle changes in gait can be an early marker of cognitive decline [10,11]. Dual-task paradigms such as the “Walking While Talking” (WWT) test highlight the cognitive demands embedded in everyday mobility [12].

Our team has established the epidemiology and risk factors associated with gait dysfunction in aging, identifying slow gait as an early precursor to dementia [10,11,13-17]. Building on this foundation, the concept of Motoric Cognitive Risk (MCR) syndrome was introduced as a predementia syndrome defined by the co-occurrence of slow gait and subjective cognitive complaints, which is associated with a high risk of developing dementia [17,18].

Our neuroimaging studies have provided insight into the neural substrates underlying gait and cognition in aging. Structural magnetic resonance imaging (MRI) studies have identified cortical and subcortical networks involved in gait control—including gray matter volume covariance networks linked to gait speed [19,20], dual-task cost during WWT [21], and MCR [19]. Functional MRI studies have identified functional brain networks associated with mental imagery of gait [22] and resting-state functional connectivity linked to actual gait performance [23]. These studies highlight that distributed brain networks support locomotion and their potential vulnerability in aging and dementia.

Advances in mobile psychophysical and neurophysiological techniques have enabled dynamic investigation of interactions among sensory, cognitive, and motor systems during or associated with real-time locomotion [24-28]. Using these approaches, studies have identified a gait-related electroencephalogram (EEG) signature in which individuals with faster gait speed exhibit sustained theta power over frontomedial regions during complex compared with simple walking [29,30]. In addition, faster gait speed [31] and better cognitive performance [32] have been linked to more efficient multisensory integration (MSI).

While average gait speed declines with age, a subset of older adults avoids this trajectory and continues to walk at speeds typical of individuals decades younger. These rare, high-functioning adults—whom we describe as Super Movers—are 80 years or older, free of dementia and disability, and maintain gait speeds comparable to adults 30 years younger [33]. Studying Super Movers offers a unique opportunity to understand mechanisms that support healthy longevity. Their preserved mobility suggests the presence of “advantageous biology,” representing a form of mobility resilience that complements other exceptional aging phenotypes. Research on centenarians [34,35], who achieve extreme longevity, and Super Agers [36], who maintain unusually strong cognitive performance, has revealed important clues about biological and behavioral resilience. Notably, Super Movers appear to be a distinct phenotype: prior work shows only modest overlap with Super Agers [33], suggesting that “moving well” and “thinking well” may rely on different biological and genetic pathways.

Accelerated biological aging and dementia-related neurodegeneration are recognized as early upstream drivers of both cognitive and motor decline [37], involving complex molecular and cellular changes that increase the brain’s susceptibility to neurodegenerative disease and hasten its overall deterioration [38]. Blood-based biomarkers have emerged as scalable markers of neurodegeneration and cognitive decline [39], but their role in gait-related cognitive processes remains relatively underexplored. Although cerebrovascular pathology is a recognized contributor to both mobility impairment [40] and cognitive decline [41], the independent contributions of neurodegenerative processes are less well-defined. Biomarkers associated with neurodegeneration, including β-amyloid and tau [42], along with genetic variants that influence individual susceptibility to pathological changes such as Apolipoprotein E (APoE) [43], have been scarcely examined in the context of biologically healthy aging, particularly in relation to gait performance and cognitive trajectories. Establishing a standardized protocol to assess Super Movers is therefore essential for understanding mobility resilience in aging and advancing early intervention strategies.

The current protocol positions Super Movers within this broad framework of exceptional aging and aims to identify factors that sustain mobility resilience and inform strategies for promoting healthy aging. We hypothesize that the interplay between protective and pathological mechanisms in aging gives rise to structural, functional, neurophysiological, and molecular brain changes that ultimately manifest as resilient gait versus gait dysfunction—a preclinical marker of dementia. By shifting attention from gait as a marker of decline to gait as a marker of exceptional mobility, this protocol leverages gait measures to elucidate mechanisms of sustained function and resilience to disease in later life.

### Study Aims

#### Aim 1: Identify Gait-Related Protective Brain Mechanisms in Super Movers:

We hypothesize that unique neural substrates and EEG activation patterns underlie exceptional gait phenotypes in older adults. To test this, we will compare structural and functional brain measures between Super Movers and usual movers aged ≥80 years using MRI, predicting that Super Movers will exhibit superior indices of brain health (Aim 1.1). We will further examine mobile EEG activation patterns during complex walking tasks, predicting that Super Movers will display distinct cortical signatures relative to usual movers of the same age (Aim 1.2). Finally, we will extend this analysis to all participants aged ≥65 years by comparing MRI and mobile EEG markers between fast movers and usual or slow movers (Aim 1.3).

#### Aim 2. Establish Neurophysiological Mechanisms of Gait and Cognitive Declines

We hypothesize that the accumulation of dementia-related pathology produces measurable functional and neurophysiological brain changes that, over time, manifest as clinically observable cognitive and gait dysfunction. To test this, differential gait-related EEG responses will be quantified using both categorical (absence of differential cortical activation) and continuous (increased variability in frontal premotor activity) metrics. We will then determine whether these differential gait-related mobile EEG responses at baseline predict longitudinal cognitive decline in adults aged ≥65 years (Aim 2.1). We will further assess whether poor baseline MSI similarly predicts cognitive decline over time (Aim 2.2). Exploratory analyses, stratified by gait status (slow, usual, and fast movers), will be conducted to identify differential neurophysiological patterns that may reflect underlying protective mechanisms. Dual cognitive-motor decline, operationalized as incident MCR syndrome, will serve as a secondary outcome.

#### Aim 3. Determine Pathological Mechanisms Underlying Gait and Cognitive Declines

We hypothesize that dementia-related brain pathology and accelerated biological aging drive the early clinical emergence of both cognitive and motor dysfunction. This aim addresses an existing gap by examining biomarkers of neurodegeneration and biological aging in relation to gait performance and cognitive trajectory. We will examine whether, among adults aged ≥65 years, slow movers exhibit greater burden of neurodegenerative pathology, characterized by elevated phosphorylated-tau (p-tau) 217, amyloid beta 40 and 42 (Aβ-40 and 42), neurofilament light chain (NfL), as well as accelerated biological aging as indexed by epigenetic clock measures [44] relative to usual movers, with fast movers hypothesized to show the inverse pattern (Aim 3.1). We will further determine whether higher baseline neurodegenerative pathology predicts longitudinal cognitive decline, and whether slow-mover status moderates the strength of this association (Aim 3.2).

## Methods

### Study Design

This study will use a mixed design combining cross-sectional and longitudinal cohort approaches. Cross-sectional analyses will examine associations between gait phenotypes, neurophysiological and neuroimaging measures, and blood-based biomarkers of both neurodegeneration and biological aging. Longitudinal follow-up will assess whether baseline biomarkers predict cognitive decline and incident MCR over time, and whether these relationships are modified by gait phenotype.

Participants will undergo brief telephone assessments every 6 months and annual in-person visits. The protocol consists of an initial telephone screening call to assess study eligibility (details given below), followed by a baseline in-person evaluation, and then subsequent biannual telephone interviews (at 6-month mark) and yearly in-house follow-up visits for up to 4 years. Blood-based biomarkers, gait measures, sensory and cognitive assessments, neurophysiologic recordings, and neuroimaging data will be integrated to characterize protective mechanisms associated with preserved mobility and cognition in aging. The study parameters are listed in Table 1 by their associated test visits.

**Table 1.** Overview of study assessments battery and measurement domains.

Study parameters	Test visit	Evaluation measures by domain
Screening	Year 1 (Telephonic interview)	Demographic (age, gender, race or ethnicity, and education) and Health Screen; T-MoCA^a^ [45]; Life space [46]
Sensory	Years 1‐3	Visual screen (Snellen test) Shoebox Auditory Test Michigan Neuropathy Screen [47,48] Pelli-Robson Odor identification test (NIH^b^ Toolbox)
MSI^d^	Years 1‐3	Visual-Somatosensory Integration Test [28] CatchU® [49]
Neurophysiological	Years 1‐3	Mobile EEG^c^ Test under 3 conditions (Normal Walking, WWT^d^, and WWMSI^e^)
Neuropsychological	Years 1‐3	5-Cog [50] Benson Complex Figure Copy and Recall [51,52] COWAT^f^ [53] CPT-3^g^ [54,55] Free and Cued Selective Reminding Test [56] Judgment of Line Test [57] Stroop Color and Word Test [58] MINT^h^ [59] Trails Making Test A and B [60] Verbal Category Switching [61] WAIS-IV^i^: Digit Span, Symbol Search, Coding [62] WRAT3^j^ [63] Wisconsin Card Sorting Test [64,65]
Neurological, medical, and physiological	Years 1‐3	Medical history (medical comorbidities, including CVD^k^) Medication and Polypharmacy List Height, weight, blood pressure, and pulse TBI^l^ history intake Brief fatigue inventory [66] Smoking and alcohol consumption intake Pittsburgh sleep quality index [67] MOS^m^ pain [68] SF-12^n^ [69]
Gait and Mobility	Years 1‐3	Quantitative gait assessment [70-72] Normal pace walking and WWT^e^ protocol [73] and Primary gait screen (Zenometrics) General mobility questionnaire
Balance, physical performance, and leisure	Years 1‐3	Unipedal stance test [74] Berg balance test [75] Biodex sensory organization test ABC^o^ scale [76] SPPB^p^ and Stair climbing [77] Grip strength; Functional reach [78] Maze [79] Leisure scale [80]
Falls	Years 1‐3	Baseline and Bimonthly Fall Interviews [72,81,82] Falls Self-Efficacy Scale [83]
Psychosocial and personality	Years 1‐3	Geriatric depression scale [84,85] Beck anxiety inventory [86] Big-5 inventory [87]
Neuroimaging	Years 1 and 3	Neuroimaging metrics: brain-predicted age (brainageR) [88] Hippocampal volume [89] Cortical thickness, resting-state fMRI^q^ [23,90-94] Structural connectivity (fractional anisotropy) [95] Cerebral small vessel disease pathologies [96]
Blood, plasma, and serum	Year 1	Complete blood count Metabolic panel Hemoglobin A1c Lipid Panel with GlycA^r^ and Diabetes Risk Index High sensitivity C-reactive protein Proteomic assay using NULISAseq Neuro 220 panel (Alamar) DNA methylation assay (Infinium Methylation EPIC v2.0).
Telephone-based cognitive and psychosocial assessment battery		
Neuropsychological	6-month Intervals	MoCA-^s^ Blind [97] HVLT^t^- R (^u^PAR) [98] WAIS-IV: digit spans, symbol search, coding [62] Oral Trail Making Test [99] Category Switching [61].

a T-MoCA: Montreal Cognitive Assessment Blind/Telephone.

b NIH: National Institutes of Health.

c EEG: electroencephalogram.

d WWT: Walking While Talking.

e WWMSI: Walking while responding to multisensory targets.

f COWAT: Controlled Oral Word Association Test.

g CPT-3: Conners Continuous Performance Test-3

h MINT: The Multilingual Naming Test.

i WAIS-IV: Wechsler Adult Intelligence Scale – Fourth Edition.

j WRAT3: Wide Range Achievement Test-3 Reading.

k CVD: cardiovascular disease.

l TBI: Traumatic brain injury.

m MOS: medical outcomes study.

n SF-12: 12-Item Short Form Survey.

o ABC: Activities-Specific Balance Confidence.

p SPPB: Short physical performance test.

q fMRI: Functional Magnetic Resonance Imaging.

r GlycA: glycoprotein acetylation.

s MOCA- Blind: Montreal Cognitive Assessment - Blind.

t HVLT-R: Hopkins Verbal Learning Test-Revised.

u PAR: Psychological Assessment Resources.

### Eligibility and Recruitment Criteria

Participants will be adults aged 65 years and older from the local communities in our catchment areas (Bronx and Suffolk counties in New York state) who speak English. Eligible individuals should be willing to participate in blood collection, EEG, mobile sensor imaging, and MRI studies. Enrollment will proceed even if any one of these procedures cannot be completed.

General exclusion criteria will include the presence of dementia, defined by a T-MoCA score <14 or documentation of a prior clinical diagnosis of dementia [45]. Terminal illness or a life expectancy of less than 12 months; progressive neurologic disorders such as amyotrophic lateral sclerosis; major psychiatric symptoms (such as psychosis) that may interfere with valid study assessment. Individuals requiring assistance from another person, mobility aids, or a wheelchair for ambulation will be excluded. Participants with metal implants or other contraindications to standard imaging procedures will also be excluded from imaging procedures.

### Recruitment Strategies

Recruitment will be strategic, using multiple complementary approaches, including electronic medical record searches within the health system (TriNetX [100]), outreach at local senior centers with exercise programs, clinician referrals, health fairs, and targeted advertisements to identify Super Movers. To support this goal, a community outreach staff member will conduct a needs assessment, collaborate with community organizations, disseminate study information, and encourage participation. These efforts will also support balanced enrollment of males and females, allowing sex as a biological variable to be examined or adjusted for in analyses.

We will recruit a total of 350 adults aged 65 years and older, consistent with our inclusion criteria. Of these, 150 participants will be newly enrolled, and about 200 will be cross-enrolled from an active sister study funded by the National Institute on Aging (Visual-somatosensory integration [VSI] as a novel marker of Alzheimer disease [AD], R01AG075679; [28]; Figure 1). We anticipate that approximately 300 participants will complete blood-based biomarker assessments. Informed consent for blood draws will be obtained from all willing participants—both newly recruited and cross-enrolled-including consent for additional biomarker assays beyond those performed in the sister cohort.

**Figure 1.**
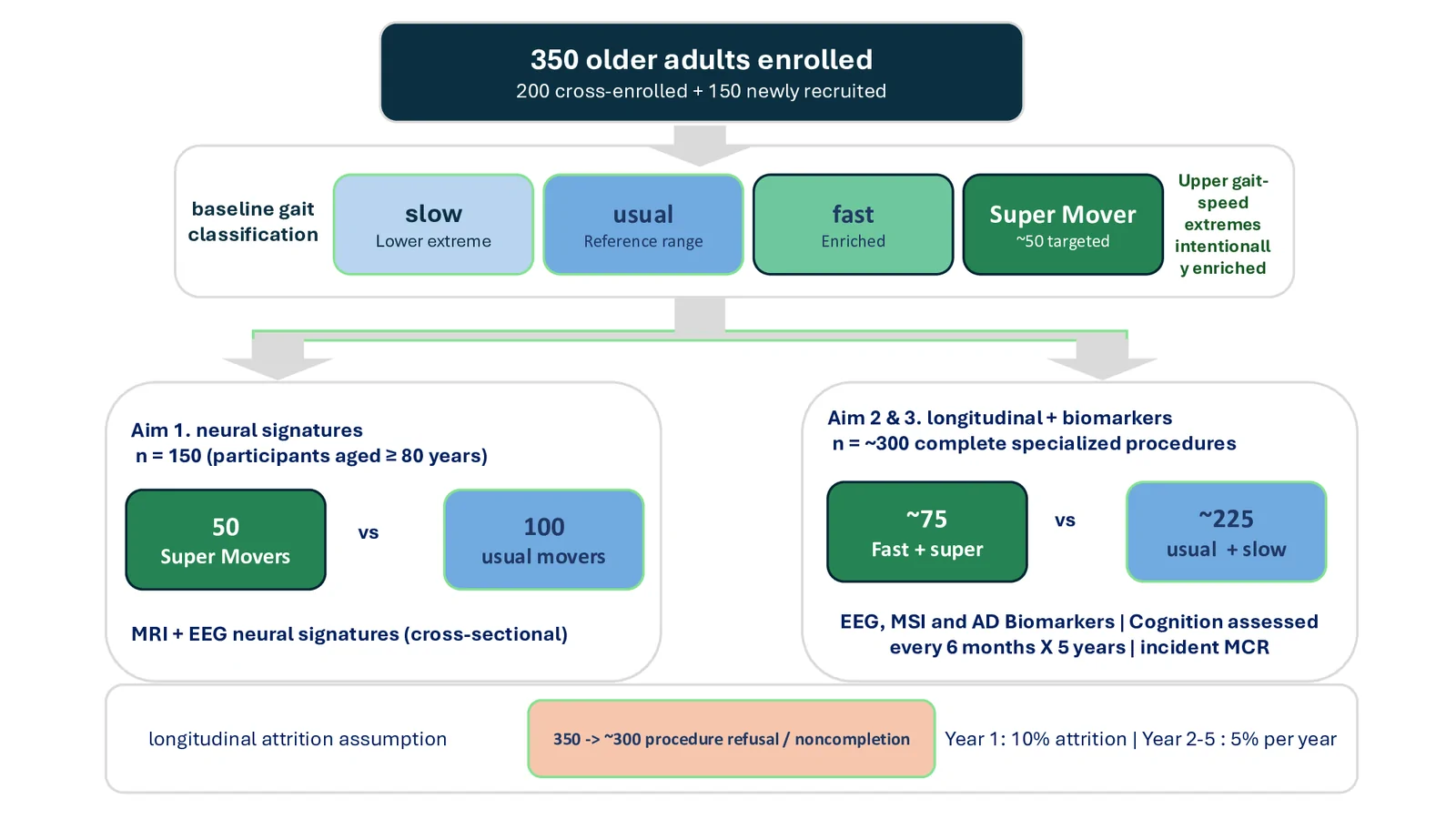
Study flow: recruitment, gait phenotype classification, and aim-specific analyzable samples. A total of 350 older adults will be enrolled (200 cross-enrolled from the parent study, R01AG075679, and 150 newly recruited), with recruitment intentionally enriched for the upper gait-speed extreme phenotypes. Aim 1 analyses (magnetic resonance imaging [MRI] and electroencephalogram [EEG] neural signatures) will be conducted in 150 participants aged ≥80 years (50 Super Movers vs 100 usual movers). Aims 2 and 3 (longitudinal cognitive outcomes, incident Motoric Cognitive Risk syndrome, and blood-based biomarkers) will include approximately 300 participants completing specialized procedures (~75 fast and Super Movers vs~225 usual and slow movers), after accounting for procedure refusal or noncompletion. Longitudinal power calculations assume 10% attrition in year 1% and 5% per year thereafter. AD: Alzheimer disease; EEG: electroencephalogram; MCR: motoric cognitive risk; MRI: magnetic resonance imaging; MSI: multisensory integration.

Potential new participants will receive an introductory email and/or letter describing the study, followed by a telephone call from trained research staff to assess interest and complete eligibility screening. Transportation will be provided for participants to attend study visits at the research center. In-person assessments will be conducted at baseline during year 1 and annually through year 3. We will aim for enrolled participants to complete all 4 baseline visits within a four-to-five-week period.

### Gait Measurements

#### Overview

Gait speed is a well-validated biomarker of aging [12] and cognitive health and is strongly associated with mobility limitations, fall risk, disability, and dementia [82]. In this study, we will implement both simple and complex dual-task walking paradigms to quantify differential neural activation, defined as complex task performance minus simple task performance. As gait depends on the integration of motor and cognitive processes, increasing task demands may enable early identification of neural vulnerability associated with dementia risk. The WWT test increases cognitive demand during walking and is sensitive to early motor and cognitive decline. Performance on this task has been shown to predict falls, mortality, and dementia [12,101,102]. We will develop quantitative gait-related EEG protocols and select gait speed as the outcome given that it is standardized, reproducible, reliable, and well-tolerated across participants [10,72,103,104].

#### Standard Gait Speed Assessment

Gait speed will be measured using two 26-foot straight instrumented walkways with embedded pressure sensors (Zenometrics, LLC) that form a track—where turning area purposefully excludes pressure sensors in order to accurately capture quantitative steady-state gait metrics. Participants will walk around the track at their usual pace during baseline and annual visits. Prior studies demonstrate excellent reliability across repeated assessments and strong predictive validity for mobility limitations, falls, and dementia [71,81,82]. The Zenometrics system captures additional spatiotemporal gait parameters that will be available for in-depth secondary analyses [10].

Gait Classification and Normative Framework

Super Movers will be defined as fast movers aged ≥80 years with a gait speed at least 1.5 SD above sex-adjusted normative cut scores [33]; this threshold corresponds to the typical gait speed of adults approximately 30 years younger. Cut scores will be derived from gait speed measured at a normal, self-selected pace across 2 straight walking segments, providing 26 feet of steady-state gait per segment. Slow movers will be defined as individuals with a gait speed at least 1.5 SD below the calculated mean based on established normative data [104,105].

### MSI Assessment

#### Behavioral MSI Task

Participants will complete an established simple reaction time (RT) task consisting of visual, somatosensory, and combined visual-somatosensory (VS) conditions, along with a catch condition in which no stimulus is presented and no response is expected [106]. Stimuli will be presented in random order with equal frequency. Each block includes 15 trials per each of the 4 conditions, and participants will complete 3 blocks for a total of 180 trials. Interstimulus intervals will vary randomly between 1‐3 seconds. Blocks will be separated by 20-second rest periods to reduce fatigue and maintain attention. This task will be conducted while sitting and once while walking on the above-noted quantitative gait mat.

Visual and somatosensory stimuli will be delivered bilaterally for a duration of 100 ms using a custom-built stimulus generator developed by Zenometrics, LLC. Stimulus triggering will be controlled using Matlab code (MATLAB R2025a, MathWorks) on a PC connected to the stimulus generator. Participants will wear headphones delivering continuous white noise to ensure somatosensory stimuli are inaudible. Participants will be instructed to respond to all stimuli as quickly as possible by pressing a pressure sensor located under their fingers.

RT from omitted trials will be set to infinity [107]. RTs outside the range of mean±2.5 SD for each stimulus type will also be set to infinity. Accuracy will be calculated as the number of correct responses divided by 45 trials per condition. Participants with task accuracy below 60% will be excluded from analyses, consistent with prior work [28].

#### Quantification of MSI

MSI magnitude will be quantified using established cumulative probability models [106]. Robust probability models will compare the combined cumulative distribution functions (CDF) of the unisensory visual and somatosensory RTs (the predicted CDF) capped at min [P(RT_V_≤ t)+ P(RTS ≤ t), 1], with the CDF of multisensory VS RTs [P(RT_VS_≤ *t*)] [28]. A violation of the race-model inequality, indicated when the observed multisensory CDF exceeds the predicted CDF, reflects MSI. The predicted CDF will be subtracted from the observed multisensory CDF to generate a CDF difference curve, and the area under the significantly violated portion of this curve will serve as the continuous measure of MSI magnitude.

#### Mobile EEG-Based Multisensory Gait Paradigm

##### Overview

As mentioned earlier and depicted in Figure 2, participants will complete overground walking tasks on a continuous instrumented walkway. Three walking conditions will be administered. During simple conditions, participants will walk at their usual pace without additional task demands. During complex conditions, participants will walk while reciting alternate letters of the alphabet and walk while responding to unisensory and multisensory stimuli, increasing cognitive and sensory load during locomotion and providing dynamic measurements of sensorimotor-cognitive interactions.

**Figure 2.**
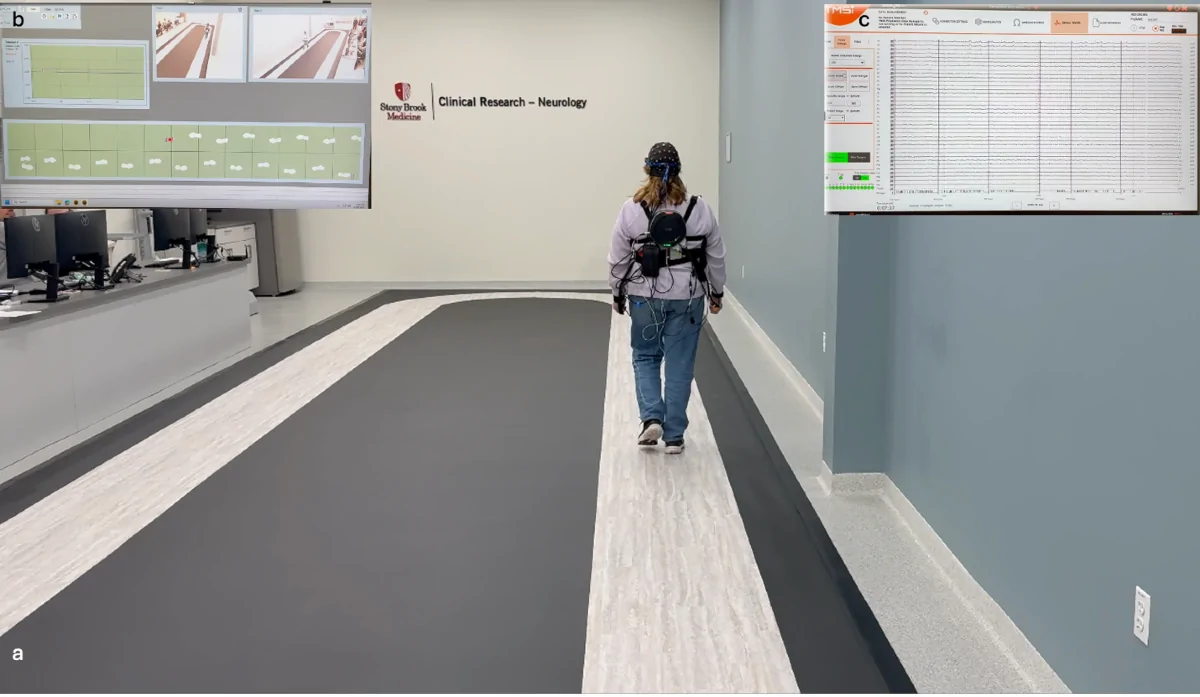
Mobile-electroencephalogram Laboratory. (A) Participant depicted walking on instrumented gait track, (B) footfalls from quantitative gait mat, and (C) raw electroencephalogram collected from electrode cap on participant’s head. Both b and c also depict time synchronized triggers.

##### Gait Analysis

Walking tasks will be presented in randomized blocks lasting approximately 5 minutes each. During complex walking blocks, participants will receive randomly presented unisensory visual, unisensory somatosensory, multisensory VS, and catch stimuli. As previously noted, all stimuli will be delivered using a custom-built multisensory apparatus (Zenometrics). Here, participants will respond with their dominant hand using handheld response devices, allowing precise measurement of reaction time and accuracy.

During these walking conditions, we will apply dynamic EEG protocols to measure oscillatory brain activity linked to the gait cycle. Evidence demonstrates a key role for brain oscillations during movement [108-110]. This approach addresses the limitations of static EEG by capturing neural dynamics during active locomotion under simple and complex conditions, enabling direct assessment of these neural patterns in an ecologically relevant context. Specifically, we will investigate neural activity during normal (self-selected pace) walking, WWT, reciting alternate letters of the alphabet [111], and while completing a multisensory simple reaction time test.

All walking blocks are performed at self-selected pace rather than at an imposed pace, since gait speed is the defining feature of the phenotypes under study. Because cortical oscillatory activity is itself modulated by walking speed [112,113], gait speed will be quantified from the instrumented walkway and modeled explicitly, as described in analysis section. Speed effects are regionally specific: when walking speed was varied parametrically to dissociate it from task difficulty, electrocortical activity changed substantially within the sensorimotor area but not in other cortical regions [114].

### EEG Acquisition and Analysis

#### EEG Recording and Preprocessing

Continuous EEG data will be recorded using a 64-channel mobile SAGA system (TMSi) at a sampling rate of 512 Hz, with a passband of 0.05 to 100 Hz and a roll-off of 24 dB per octave. Data will be high-pass filtered at 1 Hz, and notch-filtered (60 Hz) to remove line noise. Noisy channels will be identified using automated detection procedures [115] and confirmed by visual inspection prior to interpolation. Gait data will be precisely time-synchronized with EEG via transistor-transistor logic (TTL) to enable alignment of biomechanical events, neural activity, and visual attention.

#### EEG Analysis Pipeline

EEG data will be processed using a validated analysis pipeline following current guidelines for the removal of movement-induced EEG artifacts [116]. Independent component (IC) analysis will be applied to decompose signals into ICs. Resultant ICs will be localized using inverse source modeling, and only ICs with dipoles located within the brain volume and whose dipole model residual variance was below 15% will be retained. Cortical ICs will be clustered across participants [108,117].

Three analyses will be run on the retained ICs. First, log spectral power will be computed for each IC cluster and averaged across the gait cycle, providing the condition-level measure that underlies the primary Aim 1.2 outcome. Second, event-related spectral perturbations (ERSPs) will be computed from single-trial spectrograms time-locked to right heel strike, using sliding-window time–frequency decomposition normalized to the mean spectrum, to characterize gait-phase-resolved modulation in the theta, alpha, and beta bands. Third, event-related potentials (ERPs) will be extracted time-locked to the 4 VSI task conditions (unisensory visual, unisensory somatosensory, multisensory VS, and catch stimuli) to assess MSI during walking. Established guidelines for artifact rejection, component classification, and automated processing will be followed to ensure reproducibility and analytic rigor [118,119].

### Neuroimaging Measures

#### Overview

Two different resilience mechanisms will be examined: cognitive reserve and brain maintenance. Cognitive reserve is defined as preserved cognitive and physical functions despite evidence of brain changes or pathology, whereas brain maintenance refers to preserved function associated with minimal or no brain changes or detectable pathology [120]. The more general term resilience—defined as the ability to maintain cognition despite poorer brain health, function, or disease—will be used in cross-sectional examinations. Because no gold standard exists for defining protective brain morphology in aging, multimodal MRI metrics will be used to determine the presence of cognitive reserve and/or brain maintenance. These complementary frameworks will guide image selection and analytic strategy.

#### MRI Acquisition

All images will be acquired on a 3T MRI system. Image preprocessing and analyses will be conducted on a centralized Linux-based high-performance computing cluster. The following image sequences will be acquired: (1) T1-weighted structural images (3D-magnetization-prepared rapid gradient-echo [MPRAGE]), (2) diffusion-weighted images (in 66 diffusion directions), (3) 3D-fluid attenuated inversion recovery [FLAIR] imaging, and (4) T2-weighted (blood-oxygen-level-dependent [BOLD]) images during rest.

#### MRI Measures

##### Overview

The following brain metrics will be obtained from T1-weighted structural images:

1. Brain-predicted age difference (B-PAD) is a biomarker of aging generated from machine learning. Measure of brain aging is obtained by subtracting chronological age from brain-predicted age and is associated with slow gait and poor cognition [88,121-123]. Brain-predicted age is obtained from structural MRI using BrainageR because it is validated in older ages [121], but other unimodal and multimodal approaches will be considered [89,124].
2. Hippocampal volume: Overall hippocampal volume and volume of 12 different subfields will be quantified using the hippocampal tool of FreeSurfer 6.0 [91]. We reported that stride length is associated with hippocampal volume in nondemented older adults (β=0.36; *P*=.03) [125].
3. Cortical thickness: Cortical thickness (cortical gray matter) of 68 brain regions (34 per hemisphere) will be extracted with the FreeSurfer 6.0 longitudinal pipeline, which is robust to initialization points and avoids biasing towards any one time point by generating a median template for each participant based on T1-weighted images at both time points using cubic spline interpolation [126].

Exploratory analyses will examine functional connectivity in Super Movers to assess network-level associations with gait and cognition [127]. Structural connectivity will also be assessed using diffusion-weighted imaging measures of white matter integrity, including fractional anisotropy [95]. Cerebral small vessel disease markers will be included as covariates [96].

##### Neuroimaging

We hypothesize that Fast and Super Movers will exhibit slower rates of structural and functional brain change over time compared with nonfast movers (slow movers or usual movers). MRI will be performed at 24-month intervals with 100 participants, with an equal number of Fast and Super movers and comparison participants. Longitudinal analyses will examine trajectories of brain aging, with a focus on brain regions that differentiate groups at baseline.

### Blood Collection and Processing

#### Overview

Blood samples will be collected by a certified nurse practitioner within the Stony Brook University Clinical Trial Unit in Commack, New York, and processed according to standardized biospecimen handling protocols by trained study personnel. Samples will be processed and distributed promptly for laboratory analyses to minimize preanalytical variability and preserve sample integrity. Plasma and serum aliquots will be cryopreserved and banked for future investigations. Aliquots of plasma, serum, and whole blood will be transferred to Stony Brook University Freezer Farm, a centralized biorepository where samples are tracked and archived using the Freezer Works database, ensuring secure long-term storage at −80 °C for future research.

Stored serum and plasma will support a broad spectrum of biomarker analyses, including proteomic, metabolomic, and biochemical profiling. Genomic DNA will be isolated from whole blood using QIAamp DNA Blood Mini Kits from Qiagen, followed by quality assessment and quantification using the Agilent 2200 TapeStation or the Invitrogen Qubit fluorometer. Extracted DNA will be stored at -80 °C until further analysis.

Fasting will be recommended but not required. Fasting status will be recorded at the time of blood collection. Blood samples will be collected using ethylenediaminetetraacetic acid (EDTA) tubes and serum separation tubes. EDTA tubes will be used for plasma isolation and genomic DNA extraction, enabling downstream methylation array analyses for epigenetic aging clocks.

#### Blood-Based Biomarker Assessments

Blood-based biomarkers including Aβ40, Aβ42, phosphorylated (p)-tau217, and apolipoprotein E will be evaluated as indicators of neurodegeneration and amyloid pathology [43]. Selection of these biomarkers is based on prior validation studies demonstrating their relevance to AD and Alzheimer disease and related dementias (ADRD) [128,129]. NfL has emerged as a robust marker of neurodegeneration [36]. The plasma Aβ42/Aβ40 ratio, quantified using high-precision assays, has demonstrated sensitivity for detecting abnormal amyloid status in cognitively impaired and cognitively normal adults prior to amyloid positron emission tomography (PET) positivity [128,129]. Elevated baseline levels of blood p-tau, particularly p-tau217, have been shown to predict subsequent cognitive decline [130-133]. The ApoE ε4 allele is the strongest known genetic risk factor for AD, whereas the ε2 allele is a known protective factor [134].

To expand biomarker coverage within the current study, additional blood-based markers will be assessed. Serum, plasma, and whole blood samples will be analyzed for a metabolic panel, including creatinine, alkaline phosphatase, alanine aminotransferase, aspartate aminotransferase, bilirubin, hemoglobin A_1c_, a lipid panel with glycoprotein acetylation (GlycA), C-reactive protein, and complete blood count measures such as hematocrit, mean cell volume, and red cell distribution width. Samples will be shipped to Sunrise Medical Laboratories (Hicksville) for analysis. Plasma collected during the same study visit will be analyzed using the Alamar Biosciences NULISAseq Neuro 220 Panel, a multiplex proteomic assay measuring 220 biomarkers associated with neurodegenerative disease, including markers of amyloid and tau pathology, neurodegeneration, neuroinflammation, synaptic function, and vascular dysfunction [135,136].

#### DNA Methylation and Proteomic Analyses

Epigenetic aging will be assessed using DNA methylation markers. DNA methylation profiling will be performed using the Illumina Infinium HumanMethylationEPIC v.2.0 BeadChip array at the Roswell Park Comprehensive Cancer Center Genomics Shared Resource (Buffalo, NY, United States), which measures methylation at over 930,000 CpG sites across the genome.

Proteomic profiling will be conducted using the SomaScan assay from SomaLogic to explore biological pathways associated with biological aging and neurodegeneration, and to inform future mechanistic studies focused on gait phenotypes. Proteomic assessments will follow established protocols [137]. Plasma samples will be stored at -80 °C, and 130 microliter aliquots of plasma will be used to perform the SomaScan assay.

### Analysis

#### Overview

Primary hypothesis testing will be limited to prespecified primary outcomes within each aim to reduce concerns related to multiplicity. Secondary and exploratory analyses, including region-wise neuroimaging measures, EEG cluster analyses, proteomic profiling, and methylation analyses, will control the false discovery rate (FDR) within modality-specific outcome families. Analyses not designated as primary will be considered hypothesis-generating.

#### Missing Data

Two sources of missing data will be distinguished and handled separately. Modality-specific unavailability at baseline (eg, MRI contraindication, refusal, or noncompletion of a procedure) will be documented with its reason and treated as missing by design; affected participants will be excluded from analyses of that modality but retained for all aims for which their data are complete. Longitudinal missingness due to attrition or missed follow-up visits will be accommodated within the mixed-effects framework under a missing-at-random assumption. Missing data patterns will be summarized separately by source and by gait phenotype and baseline risk factors, and baseline characteristics will be compared between completers and noncompleters of each modality to assess selective unavailability.

#### Statistical Analysis for Aim 1

Descriptive statistics and graphical methods will be used to summarize data distributions and identify outliers. Transformations will be applied when needed to address skewness.

For Aim 1.1, the primary neuroimaging outcome will be brain age. Linear regression models will compare this measure between Super Movers and comparison groups, adjusting for age, sex, education, total intracranial volume, and comorbidities. Secondary neuroimaging analyses will examine additional structural and functional MRI measures using multivariate covariance-based approaches to reduce dimensionality and account for collinearity and participant variability.

For Aim 1.2, the primary EEG outcome is gait-related cortical activation, defined as the spectral power difference between complex and simple walking, averaged across the gait cycle within the a priori frontomedial theta (4‐7 Hz) independent-component cluster. The complex condition is the multisensory VS walking task, in which participants respond to unisensory and multisensory stimuli while walking. This condition is used for the primary contrast in preference to the WWT task because overt speech introduces orofacial and glossokinetic artifact that is present in the complex condition only and would therefore bias the complex-minus-simple difference. The primary comparison contrasts Super Movers with usual movers aged ≥80 years. Because Super Movers are an uncommon phenotype and group sizes may be small, the primary analysis treats this signature as a continuous measure: a linear regression of frontomedial theta activation on gait phenotype, adjusting for age, sex, education, and comorbidities (the Aim 1.1 covariate set). Effects will be reported as standardized coefficients with 95% CIs.

Gait speed: Because cortical oscillatory activity is modulated by walking speed [112,113] and gait phenotypes are defined by self-selected gait speed, speed and phenotype are partially entangled by design. The primary outcome is a within-participant difference score, with both conditions performed at the individual’s own pace, so additive person-level effects of habitual walking speed are subtracted out. Beyond this, gait speed derived from the instrumented walkway will be modeled explicitly, separating within-participant from between-participant variation, and unadjusted and speed-adjusted estimates will be reported side by side. Because gait speed constitutes the operational definition of the phenotype, complete conditioning on speed would adjust away the exposure of interest; the unadjusted estimate is therefore interpreted as the total phenotype effect and the speed-adjusted estimate as the residual component. Phenotype-by-speed interactions and dual-task-related change in walking speed will be examined, and sensorimotor alpha and beta, the bands with the most consistently documented speed dependence, will be analyzed alongside frontomedial theta as a specificity check.

Secondary analyses will (1) repeat the simple-versus-complex contrast using the WWT condition as a sensitivity analysis, with stringent independent-component removal of speech and movement artifact; (2) model presence versus absence of the differential activation signature with logistic regression, summarizing diagnostic performance using sensitivity, specificity, and area under the receiver operating characteristic curve; (3) characterize the full time-frequency response using event-related spectral perturbation across cortical IC clusters in the theta, alpha, and beta bands, with cluster-based permutation testing; (4) treat frontomedial theta variability as a continuous marker of neural stability, linking to Aim 2; and (5) examine the VS ERP measure of MSI recorded during walking. The single prespecified primary outcome limits multiplicity for confirmatory testing; all secondary EEG analyses will control the FDR within the EEG outcome family.

Longitudinal MRI data from repeat imaging in 100 participants will be analyzed using linear mixed-effects models to compare rates of brain aging over time, focusing on regions differentiating groups at baseline. Multiple comparison correction will be applied using family-wise error correction or FDR procedures, as appropriate.

#### Statistical Analysis for Aim 2

For Aim 2, the primary cognitive outcome will be longitudinal change in Hopkins Verbal Learning Test (HVLT) or Hopkins Verbal Learning Test Revised (HVLT-R) performance. Associations of baseline gait-related EEG activation patterns and MSI with cognitive decline during follow-up will be examined using linear mixed-effects models. These models account for within-participant correlation, accommodate unequally spaced observations, and handle missing outcome data under a missing-at-random assumption.

Secondary analyses will evaluate association with additional cognitive measures and MCR status using generalized mixed-effects models, and time to incident MCR will be analyzed using Cox proportional hazards models. Proportional hazards assumptions will be evaluated graphically and using scaled Schoenfeld residuals. Potential moderation by gait phenotype will be assessed by including interaction terms and by conducting stratified analyses.

#### Statistical Analysis for Aim 3

Primary analyses for Aim 3 will focus on baseline Aβ42/Aβ40 ratio, ptau217, and NfL blood-based biomarker levels that will be examined using linear regression models adjusting for relevant covariates.

Longitudinal associations of biomarkers, gait phenotype, and their interactions with cognitive decline and incident MCR will be evaluated using linear mixed-effects and Cox proportional hazards models, respectively. Similar analytic approaches will be applied to biological aging outcomes.

Secondary analyses will examine additional Alzheimer-related biomarkers. Exploratory proteomic, methylation, and mediation analyses will also be conducted to examine biological pathways linking gait phenotype, biomarkers, and cognitive decline. Mediation analyses will be exploratory and interpreted as decompositions of associations rather than definitive causal mediation unless assumptions are justified. FDR correction will be applied within biomarker modality-specific outcome families for secondary and exploratory analyses.

#### Power

As extreme gait phenotypes are uncommon in the general older adult population, recruitment will be enriched for Fast and Super Movers rather than population-proportional, with target enrollment of approximately 50 Super Movers and 50 Fast Movers; slow and usual movers will be combined for the primary analyses. For Aims 2 and 3, accounting for anticipated refusal or noncompletion of aim-specific procedures, the analyzable sample is approximately 300, comprising approximately 75 Fast and Super Movers and 225 usual and slow movers. Power calculations assume a 2-sided alpha of .05 and 80% power; for aims involving two coprimary hypotheses, a conservative 2-sided alpha of .025 was used to account for multiplicity.

For Aim 1, conducted in participants aged 80 years and older, with 50 Super Movers and 100 usual movers completing MRI, we can detect a minimum between-group difference of 0.55 SD (medium effect size) in neural signature measures, adjusting for covariates. Given the overall MRI sample of 150 in this age group and an observed 67% absence of the EEG neural signature among usual movers, we can detect an odds ratio of 0.32 for absence of the neural signature in Super Movers versus usual movers.

For Aim 2, power calculations account for unbalanced numbers of repeated measures in the longitudinal data. With 300 participants completing EEG and MSI studies, cognitive outcomes collected every 6 months over 5 years, an expected attrition rate of 10% in the first year and 5% per year thereafter, and an intraclass correlation coefficient of at least 0.5 among repeated cognitive outcomes, we can detect a difference of 0.05 SD per year in the rate of cognitive decline per 1 SD difference in baseline gait-related EEG activation pattern or MSI. With Fast and Super Movers constituting approximately 25% (75/300) of this sample, we can detect a minimum difference of 0.25 SD per year (eg, 0.5 points on the HVLT) between Fast and Super Movers and usual and slow movers in the rate of cognitive change per 1 SD difference in baseline gait-related EEG activation pattern or MSI. Based on an incident MCR rate of 51 per 1000 person-years in our local cohorts, approximately 44 incident MCR cases are expected over 5 years, providing 80% power to detect a hazard ratio of 1.62 per 1 SD increase in baseline gait-related EEG activation pattern or MSI.

For Aim 3, with 300 participants and Fast and Super Movers constituting approximately 25% of the sample by design, we can detect a between-phenotype difference of 0.43 SD (moderate effect size; two-sided alpha=.025) in blood-based biomarker levels. Under the same longitudinal assumptions as Aim 2, we can detect a difference of 0.05 SD per year in the rate of cognitive decline per 1 SD difference in baseline biomarkers, and a difference of 0.25 SD per year between Fast and Super Movers and usual and slow movers in the rate of cognitive change per 1 SD difference in baseline AD biomarkers.

Longitudinal analyses will use linear mixed-effects models, which improve statistical efficiency by incorporating repeated observations and accommodating incomplete follow-up under a missing-at-random assumption. Missing data patterns will be summarized by gait phenotype and baseline risk factors, and sensitivity analyses using multiple imputation and/or inverse probability weighting will evaluate robustness to informative missingness. Because of cross-enrollment, recruitment source will be included as a covariate or stratification factor, and baseline characteristics will be compared between newly recruited and cross-enrolled participants.

### Ethical Considerations

This study has been approved by the Stony Brook University Institutional Review Board (IRB; IRB2025-00283), and all participants will provide written informed consent. To protect privacy, each participant will be assigned a unique study ID used to link their data across all database files; the key linking IDs to names will be stored in an encrypted, password-protected master file accessible only to research staff. Identifying information will never appear in any discussion, presentation, or publication of study data. All participant data, including physical storage media, will be kept in a locked room. The database will be backed up daily, with copies stored in 2 separate locked, fire-resistant locations. Participants will be compensated up to US $350 for their time.

## Results

The Super Movers protocol was funded by the US National Institute on Aging in September 2024 as the project Gait as a Preclinical Marker for AD and Dementia: Insights from Super Movers, Usual Movers, and Slow Movers (R01AG089096)*.* The study received approval from the Stony Brook University IRB in June 2025 (IRB2025 00283). Recruitment and data collection began in August 2026, with a target enrollment of 350 participants.

## Discussion

### Anticipated Findings

Our study aims to clarify the mechanisms through which exceptional mobility contributes to resilience in late life. Preliminary observations from our group are designed to systematically evaluate whether exceptional gait performance is associated with distinct cognitive, neural, and sensory signatures. Extensive evidence linking gait performance to global and domain-specific cognition in older adults [138,139] suggests that individuals with exceptional mobility may also exhibit advantageous cognitive profiles. Notably, earlier studies [140] show minimal overlap between mobility-defined phenotypes and cognition-defined Super Agers, indicating that preserved mobility and preserved cognition may arise from partly distinct biological pathways. Whereas Super Aging has been conceptualized primarily as resistance to age-related cognitive decline within cognitive reserve and resilience frameworks [141], the Super Mover phenotype may reflect a different form of resilience grounded in sensory-motor-cognitive integration and neural efficiency [142-144]. This study will examine Super Movers as an exceptional aging phenotype in the context of cognitive reserve pathways. Longitudinal analyses across phenotypes will allow us to determine whether trajectories of mobility and cognition converge, diverge, or remain independent with advancing age.

Structural and functional neuroimaging provide complementary perspectives on the neural substrates that may support exceptional mobility. Global indices such as brain-predicted age offer an estimate of overall brain resilience and have been associated with both gait performance and cognitive aging [122]. Region-specific measures, including hippocampal volume and subfield segmentation, inform systems involved in memory and spatial navigation that are linked to mobility [145,146]. Longitudinal cortical thickness measurements provide a sensitive morphometric approach for detecting subtle, region-specific brain changes over time [147,148], while diffusion-based measures of white matter integrity and resting-state functional connectivity capture network-level organization relevant to motor and cognitive function [149,150]. Integrating these modalities with mobile EEG allows the study to test whether exceptional mobility reflects coordinated preservation across structural, functional, and dynamic neural systems.

Mobile EEG studies show that complex gait demands elicit modulation of sensorimotor beta activity, reflecting changes in motor cortical inhibition, and increases in frontomedial theta activity when cognitive control is required [151-153]. Individuals with better mobility tend to exhibit more stable and flexible recruitment of these neural systems, whereas those with poorer mobility often rely more heavily on cognitive control even during simple walking, suggesting reduced neural efficiency or early compensatory mechanisms [154]. These findings motivate the inclusion of mobile EEG in this protocol to test whether Super Movers demonstrate more consistent oscillatory modulation and lower variability in neural engagement across gait conditions. Treating gait-related neural variability as a continuous marker may reveal subtle differences in neural stability and flexibility that are not detectable through behavioral gait metrics alone. Dynamic neural recordings during real-world behaviors such as dual-task walking also capture network-level adaptations that static EEG paradigms cannot, offering insight into early vulnerability or resilience.

MSI represents an additional novel pathway through which resilience may emerge. Efficient integration of sensory inputs is essential for stable gait and adaptive movement, particularly under complex or unpredictable conditions. Prior work indicates that reduced VSI is associated with poorer gait, balance impairments, cognitive dysfunction, and greater amyloid-related pathology, supporting its potential role as an early behavioral marker of neurodegenerative processes [32,155]. Age-related declines in sensory integration have been associated with cognitive vulnerability and mobility impairments [32]. Preliminary observations suggest that individuals with exceptional mobility may demonstrate stronger MSI than their peers, even at advanced ages. The current study will evaluate whether MSI differentiates Super Movers from other mobility phenotypes and whether it predicts longitudinal trajectories of mobility and cognition. Together, these lines of evidence point to a model in which exceptional mobility in late life is supported by coordinated preservation across motor, cognitive, sensory, and neural systems.

While blood-based biomarkers are increasingly used to predict pathological aging, their role in defining trajectories of exceptional brain aging remains limited [156,157]. Most prior work has emphasized these markers as predictors of cognitive decline [158,159], with comparatively little attention to their relevance for preserving gait-related cognitive function. Our innovative study integrates blood-based biomarkers with neuroimaging, mobile neurophysiology, multisensory testing, and gait phenotyping to capture the multidimensional processes that support functional independence in aging, and anticipates addressing the existing gap in the field. This integrative framework is designed to identify mechanisms of resilience that distinguish exceptional aging from pathological trajectories. Our efforts represent an initial step toward determining whether multimodal neuroimaging, functional and neurophysiological measures, and blood-based biomarkers of neurodegeneration and biological aging can differentiate exceptional older adults from those on a pathological pathway.

### Limitations

Our study has some methodological considerations to be acknowledged, although strategies were incorporated into the design to mitigate their impact. First, because Super Movers represent a relatively uncommon phenotype in advanced aging, group sizes may differ across gait phenotypes and could reduce statistical power for categorical comparisons. To address this, neuroimaging and EEG markers will be examined as continuous measures across the full sample, and gait and cognitive trajectories will be analyzed across both phenotypes to improve statistical power. Second, all walking is performed at self-selected pace, which preserves ecological validity and the behavioral basis of the phenotype definition but means that walking speed differs between phenotypes and is itself associated with cortical oscillatory activity. Because gait speed partly defines the phenotype, speed-adjusted estimates are interpreted as a decomposition of the phenotype effect rather than as fully deconfounded estimates, and residual speed-related variance may remain. Should this prove substantial, an ancillary substudy incorporating an experimentally manipulated pace condition would be an appropriate next step. Third, although MCR syndrome and mild cognitive impairment (MCI) are independently defined constructs, some overlap between these conditions has been reported [18,103]. Accordingly, primary analyses will adjust for prevalent MCI, and sensitivity analyses will exclude participants meeting criteria for both incident MCI and MCR. Fourth, ethnoracial minorities carry a higher burden of dementia risk but remain limitedly represented in blood biomarker research, and biomarker levels may differ across groups [130]. Our study aims to include at least 40% (120/300) of participants from previous limitedly accessed populations; residual differences may still influence generalizability. These factors will therefore be considered in statistical analysis and interpretation of results.

### Conclusions

The predominant focus of aging research has normally centered on the biology of decline, identifying what goes wrong as the brain and body ages. Our protocol takes a complementary but distinct approach: by studying individuals who age exceptionally well, we aim to uncover the structural, functional, neurophysiological, and molecular brain mechanisms that sustain cognitive and motor resilience across the lifespan. Integrating state-of-the-art multimodal neuroimaging, mobile EEG, MSI, and blood-based biomarkers procedures across the full spectrum of gait performance from Super Movers to usual movers, this study is exclusively positioned to illuminate not just the pathways to dysfunction, but the pathways to resilience. Ultimately, defining the mechanisms of brain resilience may hold equal importance to identifying the mechanisms of neurodegeneration.

## Supplementary material

10.2196/104520 Peer Review Report 1 Peer review report by the National Institute on Aging Special Emphasis Panel Gait.

## Abbreviations

AD: Alzheimer disease
ADRD: Alzheimer disease and related dementias
ApoE: Apolipoprotein E
APSs: Amyloid Probability Scores
Aβ: amyloid beta
B-PAD: brain-predicted age difference
BOLD: blood-oxygen-level-dependent
CDF: cumulative distribution function
EDTA: ethylenediaminetetraacetic acid
EEG: electroencephalogram
ERP: event-related potential
ERSP: event-related spectral perturbation
FDR: false discovery rate
FLAIR: 3D-fluid attenuated inversion recovery
GlycA: glycoprotein acetylation
HVLT: Hopkins Verbal Learning Test
HVLT-R: Hopkins Verbal Learning Test-Revised
IC: independent component
IRB: Institutional Review Board
MCI: mild cognitive impairment
MCR: motoric cognitive risk
MPRAGE: 3D-magnetization-prepared rapid gradient-echo
MRI: magnetic resonance imaging
MSI: multisensory integration
NfL: neurofilament light chain
p-tau: phosphorylated-tau
PET: positron emission tomography
RT: response time
TTL: transistor-transistor logic
VS: visual-somatosensory
VSI: visual-somatosensory integration
WWT: Walking While Talking
