# Supplementary material for: An Exceptional Mobility Group Among Older Adults: Protocol for the Super Movers Study
Source: JMIR Res Protoc. 2026 Oct 30;15:e104520. doi: 10.2196/104520 (PMC13722142; doi:10.2196/104520)
Supplement: Supplementary file 1 [file resprot-v15-e104520-s001.pdf]

SUMMARY STATEMENT
( Privileged Communication )

**PROGRAM CONTACT:**
Coryse St. Hillaire-Clarke
301-496-9350
sthillaireclacn@mail.nih.gov

*Release Date:* 04/03/2024
*Revised Date:*

*Application Number:* 1R01AG089096-01

**Principal Investigator**

**VERGHESE, JOE**

**Applicant Organization:** ALBERT EINSTEIN COLLEGE OF MEDICINE

*Review Group:* ZAG1 ZIJ-G (M1)
National Institute on Aging Special Emphasis Panel
Gait as developmental AD marker

*Meeting Date:* 03/01/2024
*Council:* MAY 2024
*Requested Start:* 07/01/2024
*Opportunity Number:* RFA-AG-24-041
*PCC:* 3DMMPCS

*Project Title:* Gait as a Preclinical Marker for Alzheimer's Disease and Dementia: insights from super-movers, usual-movers, and slow-movers
*SRG Action:* Impact Score:33
*Next Steps:* Visit https://grants.nih.gov/grants/next_steps.htm
Human Subjects: 30-Human subjects involved - Certified, no SRG concerns
Animal Subjects: 10-No live vertebrate animals involved for competing appl.
Gender: 1A-Both genders, scientifically acceptable
Minority: 1A-Minorities and non-minorities, scientifically acceptable
Age: 8A-Only Older Adults, scientifically acceptable

| Project Year | Direct Costs Requested | Estimated Total Cost |
|---|---|---|
| 1 | 699,028 | 1,174,367 |
| 2 | 699,028 | 1,174,367 |
| 3 | 699,028 | 1,174,367 |
| 4 | 699,028 | 1,174,367 |
| 5 | 698,653 | 1,173,737 |
| TOTAL | 3,494,765 | 5,871,205 |

ADMINISTRATIVE BUDGET NOTE: The budget shown is the requested budget and has not been adjusted to reflect any recommendations made by reviewers. If an award is planned, the costs will be calculated by Institute grants management staff based on the recommendations outlined below in the COMMITTEE BUDGET RECOMMENDATIONS section.

**RESUME AND SUMMARY OF DISCUSSION:** This application, submitted by the Albert Einstein College of Medicine (AECoM), Bronx, New York, on behalf of Dr. Joe Verghese, the Principal Investigator (PI), requests five years of support to study the potential generation of a gait-based marker of Alzheimer's disease (AD) and Related Dementias (ADRD), leveraging three cohorts of subjects including super-, normal-, and slow-movers. This is a highly significant application trying to assess the potential role of gait abnormalities, compounded in the Multisensory Integration (MSI), as an early indicator of cognitive deterioration potentially anticipating AD/ADRD. The team of investigators, led by Dr. Verghese (gait and cognitive function expert), include cross-disciplinary and complementary expertise (Drs. Mahoney, expert on MSI and health outcomes in aging; Blumen, neuroimaging, cognition, and neuroplasticity expert; De Sanctis, mobile EEG and ambulation brain activation expert; Ayers, geriatrician and AD/ADRD clinical researcher; Sathyan, geneticist; Weiss, neuro-psychometrics; and Wang, biostatistician) has a distinguished track record in this field of study and history of collaboration. The application leverages an innovative cohort of subjects segregated by gait speed and employes cutting-edge assessments of gait, neurological function (portable/in field EEG), and neuroimaging, including novel functional MRI, for example assessing muscle oxygenation. The approach is based on strong scientific premise and proof-of-concept preliminary data, and it is organized in three independent Specific Aims (SA). Some of the proposed outcome measures are well validated and will result in robust/reliable data. The longitudinal component of the study has the potential to generate useful data to understand the chronology of gait and cognitive impairment and their correlation. The environment at AECoM is rich of academic structures, hosts a vibrant neuroscientific community, and is inserted in a resource-rich area. Shortcoming of the application includes the co-enrollment with other three studies that is complicated and makes difficult the analysis of the schedules of the measures, their rigor, and the participant burden. Furthermore, the source of the participants is not well described, their characterization for AD biomarkers is lacking, and it is unclear what other procedures or treatments participants are undergoing to. The use of super-movers could create some difficulties in the integration with the other two target groups and potentially could dilute the findings. There is lack of validation of the proposed integrated measurements of gait against more common and established ones and the sensory outcomes seem not well integrated in the research plan. SA2 and 3 lack a mechanistic exploration of the findings. Some of the paradigms proposed for the dual exploration of gait and cognition are too simplistic, for example walking while talking, and should be integrated or replaced by more representative/demanding ones. The two-year timeline to observe decline in the proposed outcome measures might be too short. Some aspects of the analytic approach need clearer justifications and the sample size is too small to support more broader generalizations. Inclusion/exclusion criteria are vaguely identified with respect to multimorbidities.

**DESCRIPTION (provided by applicant):** Emerging research implicates gait dysfunction as a preclinical marker of Alzheimer’s disease and related dementias. Our team has been in the vanguard of gait and cognition research. To advance the field, we will employ innovative gait phenotyping and multidisciplinary team science approaches to discover novel risk and protective mechanisms for gait dysfunction in the context of dementias. The aging population can be classified into slow-movers (+/- 1SD below ‘robust’ mean speed for age and sex), usual-movers (+/- 1SD) and fast-movers (≥1SD). We will employ novel multi-modal neuroscience, neurophysiological, and biological approaches to discover novel risk mechanisms in the earliest stages of gait and cognitive decline (preceding slow mover stage). We introduce the original concept of ‘super-movers’: non-demented and non-disabled fast movers, 80 years and older with gait speed of adults 30 years younger. We posit that this unique group of oldest fast movers are enriched in unique brain and biological profiles that help maintain exceptional gait into their 9th decade, and will provide a window into protective mechanisms. We hypothesize that the interplay between pathological and resilience mechanisms in aging results in structural, functional, and neurophysiological brain changes leading to the emergence of gait dysfunction as a preclinical marker of dementia. Aim 1: Identify protective brain mechanisms linked to gait in super-movers. We predict that compared to usual movers (≥80y): 1A: Super-movers will have better brain health on multi-modal measures. 1B: Super- movers will show differential activation over premotor and sensorimotor

cortex sustained across the gait cycle in response to complex gait demands. 1C: As some (but not all) fast-movers will age into super-movers, we will compare neuroimaging and mobile EEG measures in fast-movers vs usual/slow movers in all participants (≥65y). Aim 2. Establish neurophysiological mechanisms underlying gait and cognitive decline in aging. In the overall sample (≥65y): 2A: Baseline gait related mobile EEG activation patterns will predict longitudinal declines in cognition as well as combined cognitive-motor decline. 2B: Poor multisensory integration at baseline will predict longitudinal declines in cognition and Motoric Cognitive Risk syndrome. Aim 3. Determine pathological mechanisms underlying gait and cognitive decline in aging. We predict among adults ≥65y: 3A: Slow movers will have more, and faster movers will have less neurodegenerative pathology (p-tau, Aβ-42 and NFL) than usual movers. 3B: Higher levels of neurodegenerative pathology at baseline will predict cognitive decline and Motoric Cognitive Risk syndrome. This proposal is highly responsive to RFA-AG-24-041. Successfully attaining our aims will inform the use of gait as an early 'cognitive' biomarker, identify protective mechanisms that promote resilience to Alzheimer's disease and related dementias, and guide the design of early interventions to prevent cognitive decline.

**PUBLIC HEALTH RELEVANCE**: Emerging research implicates gait dysfunction as a preclinical marker of Alzheimer's disease (AD) and related dementias (ADRD). Building on our research, we will employ a team science approach with multi-modal neuroscience and biological methods to examine mechanisms underlying gait and cognitive decline in aging. Our overall hypothesis is that the interplay between pathological and resilience mechanisms in aging results in structural, functional, and neurophysiological brain changes that lead to the emergence of gait dysfunction as a preclinical marker of dementia.

**DISCLAIMER:** Please note that the following critiques were prepared by the reviewers prior to the Study Section meeting and are provided in an essentially unedited form. Even though there is opportunity for the reviewers to update or revise their written evaluation, based upon the group's discussion, there is no guarantee that individual critiques have been updated after the discussion at the meeting. Therefore, the critiques may not fully reflect the final opinions of the individual reviewers at the close of group discussion or the final majority opinion of the group. Thus, the Resume and Summary of Discussion is the final word on what the reviewers considered critical at the meeting.

**CRITIQUE 1:**

Significance: 1
Investigator(s): 1
Innovation: 3
Approach: 4
Environment: 1

**Overall Impact:**
This application will use gait as an early cognitive biomarker to identify protective mechanisms that promote resilience to ADRD using multi-modal neuroscience, neurophysiological, and biological mechanisms. The team is excellent, with a strong history and track record in this area. The application is innovative in that it proposes to look at risk and resilience collectively by proposing to recruit participants across the spectrum of movement from slow to fast to super movers. In pilot studies supporting this work, super-movers demonstrated superior cognitive and biological profiles and lower dementia risk compared to controls. The team will use mobile EEG (in laboratory) to understand brain activation in the front medial and sensorimotor cortex during ambulation and complex gait tasks. The application is innovative and timely. However, there are unaddressed gaps in the assumed underlying temporality of the brain/gait and sensory/gait connections, as often these associations can be bidirectional or dependent on heterogeneity of health status with aging. The application reads a bit crowded at times and the sensory measures seem tacked on and not well discussed. Further, the

hypotheses for SA3 are not especially novel, but understanding the pathology (or lack thereof) by movement classification is important. The approach of co-enrollment is complicated and makes it difficult to determine rigor/schedule of measures and participant burden. This is a limitation, but the infrastructure of seems to be in place to make the recruitment, testing, and retention feasible. Overall, this is a very strong application from an excellent team that is likely to shed light on novel associations in the gait/brain pathway by observing positive (super) and negative (slow) aspects of movement in conjunction with brain and cognitive health.

**1. Significance:**
**Strengths**
- A cross-disciplinary team that proposes to identify neural and non-neural mechanisms underlying gait as a preclinical marker of AD and ADRD from epidemiological, neuroscience, and neurobiological perspectives.
- Dual decline in memory and mobility affects many older adults and is not well understood. The team proposes to better identify gait and brain dysfunction and resilience to develop a preclinical marker of dementia.
- The team proposes to introduce the concept of "super-movers," who have movement profiles similar to those who are much younger, and to characterize their brain and biological profiles to identify mechanisms of resilience.
- Will use state-of-the-art gait, neurological, and neuroimaging assessments.

**Weaknesses**
- The proposed association of dementia as a mediator in the association between MSI and motor outcomes is interesting and goes against much of the popular theory that sensory impairment leads to dementia. This is important as the role of sensory impairment and integration in dementia could be as contributing causes or as concurrent signs of neurodegeneration. Although this research could help disentangle these pathways, the small sample size makes that unlikely.
- Unclear how this work may help guide the design of future interventions to prevent cognitive decline, unless interventions focus on super-movers, which are usually not targeted for intervention since they are already well functioning.

**2. Investigator(s):**
**Strengths**
- The team is interdisciplinary, with complementary expertise and history of collaboration.
- Dr. Verghese is an expert in gait and cognitive function who has pioneered much of the work in this area.
- Dr. Mahoney is an expert in multisensory integration and health outcomes in older adults.
- Dr. Blumen has expertise in neuroimaging, cognitive and neuroplasticity.
- Dr. De Sanctis is an expert in mobile EEG protocols and brain activation during ambulation.
- Dr. Wang is a Biostatistician, with extensive experience in aging research.
- Team has longstanding history of gait and cognition research.

**Weaknesses**
- Minor: Dr. Weiss's biosketch is non-specific, but her role is better explained in the budget justification.

**3. Innovation:**
**Strengths**
- The concept of super movers as a resilient profile from whom we can learn about protective gait and cognitive mechanisms is innovative.
- Incorporates underlying theories of resilience to pathology and adverse neurological processes rather than just seeking to classify "damage".
- Proposes to examine novel EEG pathways using portable technology.

**Weaknesses**

- The clinical implications of the highly technical gait measurement protocols are far removed. It would be interesting to see validation of the more complex measurement tools against more simple, clinically feasible measures of gait and function. Do the complex measures actually tell us more about the gait association between gait and cognition? However, the proposed research represents an important first step in understanding resilience to cognitive and gait decline.
- It would have been valuable to see a free-living measure of gait or physical activity to understand super-movers in their natural environment.

**4. Approach:**

**Strengths**

- Will recruit from within three active cohorts. Highly technical assessments of brain imaging (volumes) are proposed.
- The team has demonstrated expertise in the gait and neuroimaging measures proposed, with good preliminary evidence.

**Weaknesses**

- Validated cut-points for gait speed (1.0, 0.8, and 0.6 m/s) would hold more clinical utility than the cut-points based on Standard Deviation (SD) as proposed and be easier to translate clinically.
- Although normative gait values often exclude those with gait impairments, including super-movers could bias the distribution in the other direction. Specifically, if the super-movers are included in the population mean calculation, the distribution could be right-skewed and inflated. Also, exclusion criteria make it seem as though gait impairments will also be excluded here, which conflicts about normative gait populations.
- The phrase “neurophysiological neural changes” is difficult to unpack.
- General inclusion criteria A. – the bracketed text is unclear.
- The possibility of reverse causation should not be ignored – meaning, super-movers over age 80 may move better because their cognitive function allows, and not the other way around.
- It is unclear what other research is being on the participants (are there any interventions?) and whether the participants will agree to partake in another study.
- Additionally, it is difficult to understand how the overlap between studies will work. A table would help distinguish timing of measures and what is existing versus new.
- Investigators' ability to witness decline over a two-year timeframe may be limited.

**5. Environment:**

**Strengths**

- State-of-the-art facilities available for gait assessment.
- Excellent history of research support and infrastructure.
- AECoM has its own Division of Cognitive and Motor Aging, demonstrating a commitment from the institution to research in brain and motor function.

**Weaknesses**

- None noted.

**Study Timeline:**

**Strengths**

- The timeline seems acceptable given the teams access to participants in other divisional studies.
- If recruitment stays on schedule, there should be time for follow-up as proposed.

**Weaknesses**

- Limited time to see “decline” in physical and cognitive functioning.

**Protections for Human Subjects:**

Acceptable Risks and/or Adequate Protections.

- Potential risks and justifications are well described.

**Inclusion Plans:**

- Sex/Gender: Distribution justified scientifically.
- Race/Ethnicity: Distribution justified scientifically.
- Inclusion/Exclusion Based on Age: Distribution not justified scientifically.

**Budget and Period of Support:**
Recommend as Requested.

**CRITIQUE 2:**

Significance: 2
Investigator(s): 1
Innovation: 3
Approach: 6
Environment: 1

**Overall Impact:**
The goal of this project is to utilize innovative gait phenotyping and multidisciplinary team science approaches to uncover new risk and protective mechanisms related to gait dysfunction in the context of dementias. The application is based on the premise that individuals with the fastest gait, referred to as "super movers," may possess protective factors against the development of AD pathology. The underlying idea that the interplay between protective and pathological mechanisms in aging leads to neural changes resulting in gait dysfunction is robust. Furthermore, the team has contributed significantly to the field through their preliminary data. Even though the team and research environment are exceptional, there are several weaknesses in the approach. The primary concern is the lack of clear description regarding the overlap between multiple studies, including the measures collected, grant specifics, and timing of data collection. Additionally, there is a lack of specificity regarding procedures, and the rationale for collecting multiple measures that are not directly relevant to the study is unclear. These limitations ultimately reduce the impact of the application to a moderate level.

**1. Significance:**
**Strengths**

- The application addresses an important problem. Understanding the protective mechanisms that maintain gait and potentially impact dementia could lead to advances in understanding the complex relationship between motor and cognitive performance.
- There is strong evidence related to gait speed and cognition that is the foundation of this application. Much of it has been produced by this group.

**Weaknesses**

- The promise of super-movers is less compelling than understanding the slow versus usual versus fast. Understanding the protective mechanisms of fast versus slow movers seems to offer the greatest opportunity for understanding the mechanism of action and would lead to intervention development that targets those at greatest risk. This seems possible give the fast versus usual mover EEG signature data presented.

**2. Investigator(s):**
**Strengths**

- The team has a long record of accomplishments, collaboration, and skills necessary to carry out the aims of the proposed studies. They have the appropriate expertise and background for the proposed studies.

**Weaknesses**
- None noted.

**3. Innovation:**
**Strengths**
- The concept of super-movers is novel but more importantly, focusing on protective factors for normal aging and ADRD development is of interest.

**Weaknesses**
- MSI is proposed as a novel biomarker but only includes measures of cognitive and motor performance. Sensory abilities are also diminished in the preclinical pathway.

**4. Approach:**
**Strengths**
- There is benefited to increasing the size of the sample by co-enrolling participants in multiple studies.
- A longitudinal approach to examine the causal relationships between AD pathology and cognition and function will yield important insight into the development of the disease.

**Weaknesses**
- The overlap between multiple studies is not well described. What measures are collected, under which grant, at which timepoint and how does this relate to the Specific Aims? It is unclear which assessments will occur at which time and who will conduct the assessments.
- Dependence on ongoing studies as described may be a limitation. It is not clear for example, if co-recruited participants will undergo collection of plasma AD biomarkers.
- It is unclear why there are measures presented that are not germane to the aims of the study. Will these be collected on all participants? Just co-recruited participants? The burden seems significant for this set of studies.
- For SA1 identifying gait related protective mechanisms in super movers. Will MRI be prospectively collected as part of this application? Why are MRIs conducted for baseline on 150 participants and then only 100 are repeated in year 3?
- The innovative methodological approaches to multi-modal imaging to identify brain substrates of protective lack specificity with regard to how they will be characterized and analyzed.
- Some variables are underspecified (e.g., a validated community activities scale is reported for measuring the activities of the daily living, which is typically assessed in the home not in the community).
- Statistical analyses are missing important details that may impact rigor and reproducibility (e.g., no analysis specified for SA1c, unclear the assumptions of the Cox proportional hazard models).

**5. Environment:**
**Strengths**
- There are sufficient resources to conduct the studies proposed in the application.

**Weaknesses**
- It is unclear if recruitment will be achieved using the methods described.

**Protections for Human Subjects:**
Acceptable Risks and/or Adequate Protections.
- Acceptable.

**Inclusion Plans:**

- Sex/Gender: Distribution justified scientifically.
- Race/Ethnicity: Distribution justified scientifically.
- Inclusion/Exclusion Based on Age: Distribution justified scientifically.

**Budget and Period of Support:**
Recommend as Requested.
- Acceptable.

**CRITIQUE 3:**

Significance: 3
Investigator(s): 1
Innovation: 2
Approach: 4
Environment: 1

**Overall Impact:**
This application describes a project designed to investigate the phenotypes of older adults (65+ years) who are classified as slow-movers, usual-movers, fast-movers, and "super-movers" (age 80+ with gait speed of adults 30 years younger) based on age and sex norms for gait speed. The idea to explore the characteristics of "super-movers" (akin to studies of super-agers) with cognitive and motor testing, gait-related EEG profiling, neuroimaging, and blood assays is highly novel and has high potential to yield important insights into mechanisms or pathways that mediate the preservation of mobility. The PI has been highly productive in the study of the associations between Mild Cognitive Impairment (MCI), dementia, and gait impairment. The team of investigators is excellent with expertise in each of the primary experimental domains. The environment is also excellent, particularly with respect to leveraging recruitment resources available through complementary ongoing studies. For this reason, there is a highly likelihood of success and discovery. The primary strengths of the application are 1) to study participants who are categorized based on gait speed (allowing exploration of gait-related phenotyping); (2) the extensive characterization of cognitive and sensorimotor function proposed; 3) testing of gait-related cortical activation patterns with mobile EEG; and 4) the multimodal approach (behavioral, neuroimaging, neurophysiology, and blood assays). Compelling preliminary data is provided that supports the idea that super movers are a unique phenotype in terms of cognition and risk for dementia. The preliminary data on alterations in gait-related oscillations in the θ- and β-bands is interesting. There were some minor weaknesses in the design and approach of the study. Foremost of these is the plan to compare gait-related EEG measures between individuals that walk at different speeds (e.g. Figure 4 fast- versus non-fast movers) (see comments in the Approach). SA3 will examine associations between blood biomarkers of neurodegenerative pathology (phospho-tau, Amyloid-$\beta^{42}$, neurofilament light chain), gait speed and rate of cognitive decline, but will not provide mechanistic insight to the cause of the associations. Both SA2 and 3 are lacking a clear mechanistic framework or model that links the outcome measures to putative pathways contributing to the control of gait. Overall, this is a strong application with several minor weaknesses. The findings of this work, particularly derived from the extensive testing of super-movers, could have a high impact in the field.

**1. Significance:**
**Strengths**
- The experiments described in this application begin to answer an important question in the field, that is, why do some older adults continue to maintain mobility (good quality gait) and avoid cognitive decline (preservation of cognitive and motoric function; protection against risk factors). The categorization and study of individuals based on gait metrics provides a design to address this question. For this reason, the application is responsive to the RFA.

- The focus of gait-related neurophysiological biomarkers (EEG) and multisensory integration provides mechanistic insight into associations between sensorimotor metrics and cognitive function.
- The multidomain, multimodal approach is likely to yield an important database that could provide novel insights beyond the primary Aims and Hypotheses.

**Weaknesses**

- Portions of SA2 and 3 are designed to explore associations between the gait and cognitive metrics but will not provide novel mechanistic insight underlying the associations.

**2. Investigator(s):**

**Strengths**

- Excellent interdisciplinary team of investigators.
- The PI has demonstrated extensive productivity in the research focused on associations between risk factors for cognitive decline, MCI, dementia, and alterations in gait.
- The research team includes individuals with expertise and demonstrated productivity in neuroimaging (Dr. Blumen), mobile gait-related EEG (De Sanctis), neuro-psychometrics (Drs. Mahoney and Weiss) and biostatistics (Dr. Wang)

**Weaknesses**

- None noted.

**3. Innovation:**

**Strengths**

- To study “super-movers” is highly innovative, potentially providing insight into protective and/or preservation of function mechanisms.
- The use of portable EEG to study gait-related cortical activity under normal and visual perturbed conditions is novel and the preliminary data is compelling.
- Categorization of the cohort into groups based on “robust” norms.
- Study of MSI and its relationship to cognition and gait.

**Weaknesses**

- The MRI methods and outcome measures are not particularly novel and weakly linked to gait-related function.
- The blood assay approach to examine associations to cognitive and gait metrics is not novel.

**4. Approach:**

**Strengths**

- Categorization of the participants based on gait speed norms and identification of super movers is a major strength (albeit this categorization should be based on normalized gait speed; e.g., statures, due to alterations in stature with aging).
- The gait-related EEG protocol is well developed (valid, reliable) and will provide metrics than could provide meaningful insight into the mechanisms of slowed gait in people with cognitive impairment.
- Table 2: Battery of tests seems thorough, well thought out, and appropriate.

**Weaknesses**

- A concern that could confound the results is the plan to compare gait-related EEG measures between individuals that walk at different speeds. Although this is a good method to categorize participants, the primary outcome measures need to be compared both at natural speed and matched speeds (normalized to stature). The profiles of cortical oscillations are affected by gait speed. Thus, it is plausible that the EEG profile of slow movers could resemble that of fast movers if they simply speed up, or vice versa if fast movers walking slower.
- Many of the references are out of context with the text (e.g. refs 8-10 on Aims page)

- The term “better brain health” is used throughout the application. This term is vague, poorly defined, and does not provide mechanistic insight since is it predominantly used on the context of correlations (associations) with higher cognitive or motor function.
- Walking while talking as a dual-task paradigm seems a little weak; there are other protocols that might be more sensitive to cognitive demand during walking.
- The use Brainage R to drive metrics of predicted biological age is weak. The accuracy/validity of this approach when studying within an older cohort, is not sufficient.
- The longitudinal design of the study was not well described. The SAs pages states that metrics will be obtained every six months for five years, but the timeline describes longitudinal follow-up for a “minimum of two years”. What measures are collected at what timepoints?
- The MSI task was not sufficiently described.

**5. Environment:**
**Strengths**
- Excellent environment that has the resources necessary to accomplish the SAs.
- Will leverage the resources and recruitment infrastructure associated with ongoing complementary studies aging, gait, and cognition. The group has demonstrated success in recruitment of large numbers of participants.

**Weaknesses**
- None noted.

**Protections for Human Subjects:**
Acceptable Risks and/or Adequate Protections.

**Data and Safety Monitoring Plan:**
Acceptable.

**Inclusion Plans:**
- Sex/Gender: Distribution justified scientifically.
- Race/Ethnicity: Distribution justified scientifically.
- Inclusion/Exclusion Based on Age: Distribution justified scientifically.

**Resource Sharing Plans:**
Acceptable.

**Budget and Period of Support:**
Recommend as Requested.

**THE FOLLOWING SECTIONS WERE PREPARED BY THE SCIENTIFIC REVIEW OFFICER TO SUMMARIZE THE OUTCOME OF DISCUSSIONS OF THE REVIEW COMMITTEE, OR REVIEWERS' WRITTEN CRITIQUES, ON THE FOLLOWING ISSUES:**

**PROTECTION OF HUMAN SUBJECTS: ACCEPTABLE.** No concerns were reported by the scientific review group pertaining to the compliance with the NIH regulations for the protection of human subjects.

**INCLUSION OF WOMEN PLAN: ACCEPTABLE.** No concerns were reported by the scientific review group regarding the enrollment of women.

**INCLUSION OF MINORITIES PLAN: ACCEPTABLE.** No concerns were reported by the scientific review group in relation to minority groups' recruitment.

**INCLUSION ACROSS THE LIFESPAN: ACCEPTABLE.** No concerns were reported by the scientific review group regarding the inclusion throughout the lifespan; children exclusion is scientifically justified.

**COMMITTEE BUDGET RECOMMENDATIONS: The budget was recommended as requested.**

---

Footnotes for 1R01AG089096-01; PI Name: VERGHESE, JOE

NIH has modified its policy regarding the receipt of resubmissions (amended applications).See Guide Notice NOT-OD-18-197 at https://grants.nih.gov/grants/guide/notice-files/NOT-OD-18-197.html.  The impact/priority score is calculated after discussion of an application by averaging the overall scores (1-9) given by all voting reviewers on the committee and multiplying by 10. The criterion scores are submitted prior to the meeting by the individual reviewers assigned to an application, and are not discussed specifically at the review meeting or calculated into the overall impact score. Some applications also receive a percentile ranking. For details on the review process, see http://grants.nih.gov/grants/peer_review_process.htm#scoring.

**MEETING ROSTER**

The roster for this review meeting is displayed as an aggregated roster that includes reviewers from multiple AG Special Emphasis Panels of the NIA SEP Aggregate roster for May 2024 Council for the 2024/05 council round.
This roster for AG is available here.
